# Use of the creating opportunities for parent empowerment programme to decrease mental health problems in Ugandan children surviving severe malaria: a randomized controlled trial

**DOI:** 10.1186/s12936-021-03795-y

**Published:** 2021-06-13

**Authors:** Paul Bangirana, Annet Birabwa, Mary Nyakato, Ann J. Nakitende, Maria Kroupina, John M. Ssenkusu, Noeline Nakasujja, Seggane Musisi, Chandy C. John, Richard Idro

**Affiliations:** 1 Department of Psychiatry, Makerere University College of Health Sciences, Kampala, Uganda; 2 Department of Mental Health and Community Psychology, Makerere University College of Humanities and Social Sciences, Kampala, Uganda; 3 Department of Pediatrics, University of Minnesota, Minneapolis, MN USA; 4 Department of Epidemiology and Biostatistics, Makerere University College of Health Sciences, Kampala, Uganda; 5 Ryan White Center for Pediatric Infectious Disease and Global Health, Indiana University, Indianapolis, IN USA; 6 Department of Pediatrics and Child Health, Makerere University College of Health Sciences, Kampala, Uganda

**Keywords:** Severe malaria, Behavioural problems, Mental health, Caregiver training

## Abstract

**Background:**

Severe malaria is associated with long-term mental health problems in Ugandan children. This study investigated the effect of a behavioural intervention for caregivers of children admitted with severe malaria, on the children’s mental health outcomes 6 months after discharge.

**Methods:**

This randomized controlled trial was conducted at Naguru Hospital in Kampala, Uganda from January 2018 to July 2019. Caregiver and child dyads were randomly assigned to either a psycho-educational arm providing information about hospital procedures during admission (control group), or to a behavioural arm providing information about the child’s possible emotions and behaviour during and after admission, and providing age appropriate games for the caregiver and child (intervention group). Pre- and post-intervention assessments for caregiver anxiety and depression (Hopkins Symptom Checklist) and child mental health problems (Strength and Difficulties Questionnaire and the Child Behaviour Checklist) were done during admission and 6 months after discharge, respectively. T-tests, analysis of covariance, Chi-Square, and generalized estimating equations were used to compare outcomes between the two treatment arms.

**Results:**

There were 120 caregiver-child dyads recruited at baseline with children aged 1.45 to 4.89 years (mean age 2.85 years, SD = 1.01). The intervention and control groups had similar sociodemographic, clinical and behavioural characteristics at baseline. Caregiver depression at baseline, mother’s education and female child were associated with behavioural problems in the child at baseline (p < 0.05). At 6 months follow-up, there was no difference in the frequency of behavioural problems between the groups (6.8% vs. 10% in intervention *vs* control groups, respectively, p = 0.72). Caregiver depression and anxiety scores between the treatment arms did not differ at 6 months follow-up.

**Conclusion:**

This behavioural intervention for caregivers and their children admitted with severe malaria had no effect on the child’s mental health outcomes at 6 months. Further studies need to develop interventions for mental health problems after severe malaria in children with longer follow-up time.

*Trail registration* ClinicalTrials.gov Identifier: NCT03432039

**Supplementary Information:**

The online version contains supplementary material available at 10.1186/s12936-021-03795-y.

## Background

Malaria remains one of the leading causes of morbidity globally with 219 million cases reported in 2017 with over 90% in sub-Saharan Africa [1]. Some of these cases are severe leading to death especially in children under 5 years old in Africa [1]. With more effective treatment for severe malaria, more children now survive the disease than before [2]. Recent studies have shown that children surviving severe malaria have behavioural problems up to 24 months post discharge [3, 4]. These include internalizing (depression and anxiety) and externalizing problems (attention deficit hyperactivity disorder, oppositional defiant disorder and conduct disorder) [3, 4]. Behavioural problems in childhood are associated with future psychiatric complications, challenges with education, employment, and social life [5, 6]. In Ugandan children, behavioural problems after severe malaria may lead to harsh punishments from caregivers as a way of making a child behave well [7].

Children admitted to intensive care units (ICU), such as those with severe malaria, are exposed to stressors, such as invasive procedures, respiratory insufficiency, delirium with possible psychotic experiences, different professionals providing care, and separation from families leading to mental health problems [8, 9]. As a result of a traumatic ICU experience, post-traumatic stress disorder (PTSD) is common in children, followed by depression after admission for a life-threatening illness [8]. Interventions aimed at preventing these psychological reactions after discharge should address the child and caregivers’ experiences of these stressors on the ward.

Creating opportunities for parent empowerment (COPE) is an educational and behavioural intervention for children admitted to ICU and their caregivers to prevent adverse psychological reactions [10]. It creates a sense of control in the parent while on the ward through simple play activities that a parent and child can engage in and follows up the family after discharge to explain the likely post-discharge emotional problems and what parental behaviours can help to reduce these problems [10]. COPE postulates that information given to caregivers helps them anticipate what will occur on the ward and develop problem-focused coping techniques to deal with the situation [10]. The activities through which the parent engages with the child instil feelings of control, thus lessening anxiety. They also create an environment where negative emotions are controlled reducing the likelihood of them being transferred to the child.

The present study evaluated the effect of this educational and behavioural intervention for caregivers and their children admitted with severe malaria. Caregivers and their children were assessed for mental health problems at baseline prior to the intervention and 6 months after discharge from hospital.

## Methods

### Study design and participants

This was a randomized, controlled trial where children were assigned 1:1 to either a behavioural or psycho-educational treatment. Participants were children aged 1.5 to 4 years. The inclusion criteria were: (a) aged 1.5 to 4 years; (b) admitted with severe malaria necessitating admission and intravenous anti-malarial medication; and, (c) signed informed consent from the caregiver. Severe malaria in this study included: cerebral malaria, severe malarial anaemia, malaria with impaired consciousness (but not in coma or cerebral malaria), and malaria with multiple seizures. The exclusion criteria were: (a) living more than 50 km from the hospital; and, (b) pre-existing developmental delays based on the Ten Questions Questionnaire [11].

### Study site

The study was conducted at Naguru General Hospital in Kampala city, the capital of Uganda. This site was chosen because of its large catchment area, which enabled the study to obtain a fairly representative sample of Kampala and its surroundings.

### Interventions

#### Behavioural intervention (experimental group)

This intervention was a modified version of the original COPE programme [12]. This is a behavioural intervention that educates the parent about the children's likely emotional and behavioural problems that may result from admission for a critical illness. It provides the parent with skills to deal with these problems and bring about a change in the child's behaviour as outlined below. This intervention (as well as the control intervention) was delivered by a graduate-level psychologist who was not involved in assessment of the study outcomes. The COPE intervention was delivered in three phases with Phase I being delivered within 6 to 16 h of admission to the hospital where caregivers were provided with information about the child's likely emotional reactions during admission in hospital (see Additional file 2; Intervention script). Phase II was delivered within 2 to 16 h of transfer to the general ward and consisted of: (a) verbal and written information to reinforce information provided in Phase I plus additional information on children’s responses during and following hospitalization, as well as to provide further suggestions to enhance coping outcomes in their children; and, (b) parent–child skills-building activities. This consisted of three activities to be completed before discharge from the hospital: (i) doll play to encourage expression of emotions in a non-threatening manner; (ii) therapeutic medical play to assist children in obtaining some sense of mastery and control over the hospital experience; and, (iii) telling a story about a young child who successfully copes with a stressful hospital admission. Parents were encouraged to engage their children in these games thereafter during admission. The modification in this study involved removing audio-taped instructions from Phases I and II, as in the original study, and instead having the intervention delivered face-to-face [12].

Phase III of the behavioural intervention programme occurred 2 to 3 days after hospital discharge and consisted of a telephone call during which a 5-min script was read that reinforced the following: (a) young children’s typical post-discharge emotions and behaviours; and, (b) parenting behaviours which would continue to facilitate positive coping outcomes in their children. Mothers were encouraged to continue performing the activities from Phase II that they received during hospitalization.

#### Psycho-education intervention (control group)

This intervention also had three phases occurring at the same time as the behavioural intervention [12]. Phase I provided verbal and written information about the paediatric admission unit services and policies. Phase II consisted of: (a) verbal and written information about the general paediatric unit and its policies; and, (b) a parent–child activity having ‘control’ activities, such as reading a story not related to hospital stay. Phase III of the control programme consisted of a telephone call 2–3 days after discharge during which mothers were informed that they should contact their primary healthcare providers if their children were having any problems or unusual symptoms. They were also asked to comment on their children’s hospital stays during this telephone call (see Additional file 1; control script).

The games and stories of the interventions were different for the age groups. The hospital in which the study was conducted has an open general ward for children, which made it impossible to separate participants from the different arms while on the ward to prevent them from observing different games and activities of the other intervention.

### Outcomes

Primary and secondary outcomes were assessed during admission prior to the intervention and at 6 months after discharge. Presence of a behavioural problem was the primary outcome of the study, which was assessed using the self report Strengths and Difficulties Questionnaire (SDQ) [13, 14]. It has 25 items assessing five domains of five items each: emotional, conduct, hyperactivity, peer, and prosocial problems. Summation of scores from the first four scales gives the total difficulties score, which was the primary outcome measure for the SDQ [14]. The SDQ has been used in Uganda to screen for behavioural problems, including a study on children with severe malaria and has proven a valid measure in this region with a sensitivity of 60% when compared with a diagnostic interview [3, 15–17]. In the present study, the internal reliability of the SDQ was 0.60. A score of 17 or more was indicative of behavioural problems.

Total behavioural problems score in the children were assessed using the preschool version of the Child Behaviour Checklist (CBCL) [18], which was a secondary outcome of the study. The CBCL has 100 items about a child’s behaviour that the parent responds to, which can be summarized into seven sub-scales which are further summarized into externalizing, internalizing and total problems [19]. The CBCL has been used in several studies in Uganda and is reliable in assessing behaviour over time with test–retest reliabilities for its scales ranging from 0.64 to 0.83 [4, 20]. The CBCL has not been compared with a structured clinical interview for validation in the study’s setting. It was included to supplement the SDQ, given its broad assessment of behavioural problems using its widely used syndrome scales [21].

Maternal depression and anxiety were secondary outcomes which were assessed using the 25-item version of the Hopkins Symptom Checklist (HSCL) [22]. Anxiety and depression are common outcomes in parents whose children have been in ICU [23]. The HSCL has 25 items with the first 10 assessing anxiety and the next 15 assessing depression. Its reliability ranges from 0.83 to 0.91 for the different subscales and has a sensitivity of 81% in Ugandan adults [24].

A socio-economic status form used in previous studies in Uganda was used to measure the material possessions of the family, housing type, cooking resources and water source [25, 26]. These were scored and summed up to obtain a socio-economic status score. The Ten Questions questionnaire [11, 27] was used to screen for children with neurodevelopmental delay who could have pre-existing behavioural problems that could confound the intervention outcomes. It is a widely used screen for neurodevelopmental disabilities used in a field survey of neurodevelopmental disabilities in Uganda [28].

### Sample size estimation

In the original COPE trial, an absolute difference of 23.6% in the prevalence of behavioural problems between the control and COPE arms (25.9% vs. 2.3%, respectively) was observed 12 months after the intervention [12]. In the proposed study, the 6-month assessment was the primary endpoint. Assuming the COPE arm to have 2.3% with behavioural problems [12] and the control arm would have the same rate as observed in Idro et al. (18.5%) [3], a sample size of 55 per group was needed for a study powered at 80%. Assuming a loss to follow-up of 10%, 60 children per arm were targeted for enrolment.

### Randomization procedure

Stratified randomization was done by age groups, i.e., 1 year old band, 2 years old band, 3 years old band, and 4 years old band were randomized individually. For each of these age groups, random numbers were computer generated by the first author and the treatment allocation kept in sealed opaque envelopes serially numbered to conceal allocation. The psychologist administering the intervention had custody of these envelopes, which she opened to reveal the treatment group once a participant was enrolled by the study nurse. Assessors of the child’s mental health were blinded to the child’s treatment arm allocation by not involving them in providing the intervention or access to group allocation envelopes. Caregivers were also blinded to the child’s treatment allocation, however because it was impossible to separate them on the ward, there is a possibility they may have observed different interventions being given to other children.

### Data management and analysis

Data were entered into FileMaker with validation checks and exported to IBM SPSS Statistics for Windows, version 26 (IBM Corp., Armonk, NY, USA) for analysis. The Chi square test was used to compare the rates of children with a behavioural problem between the two groups. T-tests were used to compare continuous scores between the study groups. Analysis of covariance controlling for maternal anxiety and child’s gender was used to compare 6-month behavioural problems in the treatment arms. To account for multiple variables, outcome measurements were compared between the intervention and control groups using generalized estimating equations (GEE) with robust standard errors and assuming an exchangeable correlation structure between the measurements at the two-time points.

## Results

### Participant recruitment

**Fig. 1:**
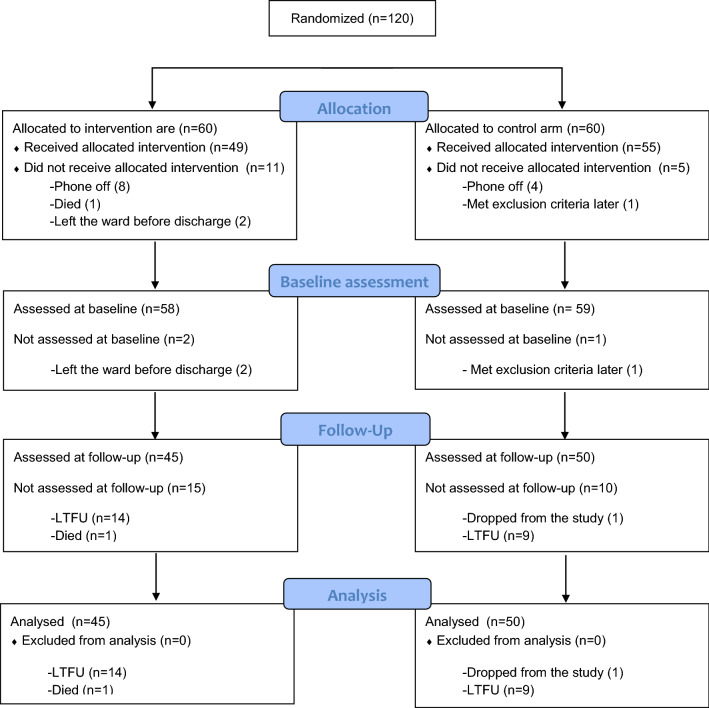
Study flow diagram

One-hundred and twenty participants were recruited into the study from January to November 2018 and followed up 6 months later from June 2018 to July 2019. Sixty were assigned to the control arm of which 55 received the intervention and 50 were analysed for the primary outcome. Sixty were assigned to the intervention arm, 49 received the intervention and 45 were analysed for the primary outcome. The study profile (Fig. 1) provides details of the numbers that were excluded, leading to the final numbers assessed. The study concluded when the last participant who could be located was assessed after 6 months.

### Baseline characteristics of the study participants

The mean age of the children was 2.85 years (SD = 1.10) with 51.7% female. Children in both treatment arms had similar sociodemographic, clinical and behavioural characteristics (Table 1). Baseline behavioural problems in children were associated with caregiver depression, caregiver education and child’s gender (Table 2). Caregiver’s anxiety and depression at baseline during admission were associated with presence of diarrhoea and behavioural problems in the child.

**Table 1:** Baseline characteristics of the study participants

Characteristic	Intervention (n = 59)	Control (n = 59)	P value
Age, years	2.85 (1.01)	2.84 (1.03)	0.96
Sex, female (n, %)	31 (52.5%)	30 (50.8%)	0.85
Socioeconomic status score	11.75 (3.73)	12.85 (2.81)	0.08
Child in school (n, %)	15 (26.3%)	17 (28.8%)	0.76
Mother’s education (n, %)			0.85
None	2 (3.6%)	4 (7.0%)	
Primary	23 (41.8%)	22 (38.6%)	
Secondary	27 (49.1)	27(47.4%)	
Tertiary	3 (5.5%)	4 (7.0%)	
Father's education (n, %)			0.19
None	1 (1.9%)	1 (1.8%)	
Primary	16 (29.6%)	13 (22.8%)	
Secondary	26 (48.1%)	38 66.7%)	
Tertiary	11 (20.4%)	5 (8.8%)	
Weight for age z score	− 0.80 (1.07)	− 0.56 (1.06)	0.22
Temperature, °C	38.56 (1.23)	38.66 (0.88)	0.61
Days with fever	4.88 (3.55)	4.72 (3.73)	0.81
Coughing (n, %)	35 (59.3%)	40 (69.0%)	0.28
Difficulty breathing (n, %)	12 (20.3%)	6 (10.3%)	0.13
Diarrhea (n, %)	14 (23.7%)	18 (31.0%)	0.38
Vomiting (n, %)	29 (49.2%)	26 (44.8%)	0.64
Convulsions (n, %)	20 (33.9%)	15 (26.3%)	0.37
Last fed, hours	26.72 (30.56)	22.69 (28.32)	0.48
Last drank, hours	3.09 (7.86)	2.40 (2.89)	0.56
Respiratory rate	38.70 (12.96)	34.58 (12.51)	0.24
SDQ behavioral problem in the child (n, %)	23 (40.4%)	20 (33.9%)	0.47
SDQ total score	15.47 (5.18)	15.56 (4.57)	0.93
CBLC total problems	− 0.83 (0.82)	− 0.91 (0.83)	0.58
CBLC internalizing problems	− 1.08 (0.64)	− 1.12 (0.66)	0.73
CBLC externalizing problems	− 1.19 (0.58)	− 1.27 (0.66)	0.51
HSCL caregiver anxiety score	5.75 (5.49)	6.00 (6.17)	0.82
HSCL caregiver depression score	12.72 (9.39)	12.64 (9.70)	0.97

All figures are mean (standard deviation) unless otherwise stated. Frequencies compared with Chi squared testing. Continuous values compared with Students t-test

**Table 2:** Risk factors for behavioral problems in children at baseline

Characteristic	Behavioral problem^a^ (n = 43)	No behavioral problem (n = 73)	P value
Age, years	2.82 (1.03)	2.88 (1.02)	0.79
Sex, female (n, %)	27 (62.8%)	32 (43.8%)	**0.05**
Socioeconomic status score	12.47 (3.32)	12.22 (3.35)	0.70
Child in school (n, %)	11 (25.6%)	21 (28.8%)	0.71
Mother’s education (n, %)			**0.05**
None	2 (4.9%)	4 (5.6%)	
Primary	22 (53.7%)	23 (32.4%)	
Secondary	13 (31.7%)	41 (57.7%)	
Tertiary	4 (9.8%)	3 (4.2%)	
Father's education (n, %)			0.99
None	1 (2.3%)	1 (1.5%)	
Primary	11 (25.6%)	18 (26.5%)	
Secondary	25 (58.1%)	39 (57.4%)	
Tertiary	6 (14.0%)	10 (14.7%)	
Weight for age z score	− 0.80 (1.12)	− 0.62 (1.03)	0.38
Temperature, °C	38.77 (1.04)	38.50 (1.08)	0.19
Days with fever	4.67 (3.13)	4.93 (3.91)	0.71
Coughing (n, %)	28 (66.7%)	46 (63.0%)	0.69
Difficulty breathing (n, %)	7 (16.7%)	11 (15.1%)	0.82
Diarrhea (n, %)	15 (35.7%)	17 (23.3%)	0.15
Vomiting (n, %)	21 (50.0%)	34 (46.6%)	0.72
Convulsions (n, %)	11 (26.2%)	22 (30.6%)	0.62
Last fed, hours	25.03 (30.20)	24.12 (28.85)	0.88
Last drank, hours	2.00 (2.09)	3.10 (6.80)	0.41
Respiratory rate	40.24 (14.94)	34.23 (10.94)	0.10
Caregiver anxiety score	7.12 (6.30)	5.15 (5.44)	0.08
Caregiver depression score	15.40 (10.62)	11.08 (8.47)	**0.02**

All figures are mean (standard deviation) unless otherwise stated. Frequencies compared with Chi squared testing. Continuous values compared with Students t-test

^a^An SDQ score ≥ 17

### Child and caregiver behavioural outcomes at 6-month follow-up

The frequency of behavioural problems in the intervention arm (6.8%) versus the control arm (10%) was not different (relative risk 0.66, 95% CI 0.15–2.93, p = 0.72). There were no differences between the treatment arms in the secondary outcomes from both the SDQ and CBCL at 6 months follow-up after controlling for baseline caregiver depression, and education level (Table 3). Similarly, there were no differences in caregiver anxiety and depression outcomes at 6 months after controlling for presence of diarrhoea and behavioural problems in the child. GEE models accounting for multiple variables also showed no differences in child and parental outcomes between the groups (see Additional file 3: Table S1).

**Table 3:** Child and caregiver emotional and behavioral outcomes at 6 months

Domain	Intervention (n = 45)	Control (n = 50)	Mean difference (95% CI)	P value
Behavioral problem in the child (n, %)	3 (6.8%)	5 (10.0%)		0.72
SDQ total problems	10.26 (0.71)	10.69 (0.66)	− 0.43 (− 2.35 to 1.50)	0.66^a^
SDQ emotional problems	2.23 (0.29)	2.22 (0.27)	0.01 (− 0.79 to 0.81)	0.99^a^
SDQ conduct problems	2.21 (0.29)	2.98 (0.27)	− 0.77 (− 1.55 to 0.01)	0.054^a^
SDQ hyperactivity problems	3.99 (0.29)	3.76 (0.27)	0.22 (− 0.57 to 1.02)	0.58^a^
SDQ peer problems	1.84 (0.22)	1.72 (0.20)	0.12 (− 0.47 to 0.70)	0.70^a^
SDQ prosocial problems	7.78 (0.25)	8.15 (0.24)	− 0.37 (− 1.06 to 0.32)	0.29^a^
CBCL total problems	− 1.38 (0.12)	− 1.32 (0.12)	− 0.06 (− 0.39 to 0.27)	0.74^a^
CBCL internalizing problems	− 1.20 (0.10)	− 1.20 (0.10)	0.002 (− 0.27 to 0.28)	0.99^a^
CBCL externalizing problems	− 1.35 (0.12)	− 1.17 (0.11)	− 0.18 (− 0.50 to 0.14)	0.26^a^
HSCL caregiver anxiety	2.94 (0.69)	4.51 (0.66)	− 1.57 (− 3.46 to 0.32)	0.10^b^
HSCL caregiver depression	8.85 (1.14)	9.24 (1.09)	− 0.38 (− 3.53 to 2.77)	0.81^b^

All figures are mean (standard error) unless otherwise stated. Frequencies compared with Fisher’s exact test. Continuous values compared with analysis of covariance

^a^Adjusted for caregiver depression, mother’s education and child’s sex

^b^Adjusted for presence of diarrhea and behavioral problems for the child during admission

## Discussion

This study set out to examine the effect of a caregiver behavioural intervention for children admitted with severe malaria to prevent mental health problems 6 months after discharge. There were no differences in mental health outcomes between the two groups after 6 months. Mental health problems in the children during admission were associated with caregiver depression, caregiver education and child’s gender.

Admission for children in hospitals can be a stressful experience and is associated with anxiety and depression in caregivers and children in both the acute period of the disease and in the long-term period [8, 9]. Ugandan children with severe malaria have mental health problems in the short and long-term, including hyperactivity, aggression and mood changes [3, 4]. Studies show that mental health problems in children admitted in hospital are associated with illness severity, duration of admission and pre-morbid mental health problems [29, 30]. In the present study, only caregiver depression, caregiver education and child’s gender were associated with the child’s mental health problems during admission. However disease severity in terms of having diarrhoea during admission was associated with caregiver anxiety and depression scores. Caregivers of admitted children feel out of control, leading to anxiety which can be transferred onto the child who sees the caregiver in that state [10]. The present study found a correlation between caregivers’ anxiety and depression scores and the child’s mental health scores, which is in line with the emotional contagion theory [10].

In the present study, there was a higher prevalence of behavioural problems among females. Prior studies in Uganda using the SDQ and CBCL, that were used in the present study, found no association between gender and behavioural problems [3, 20]. Idro et al. observed a non-significant trend of females having a history of severe malaria being more likely to have internalizing problems compared to males who had externalizing problems [3]. The observation of an association between females and behavioural problems in the present study compared to others could be due to the timing of assessments. The present study’s assessments were done during admission compared to post-discharge in the previous studies.

The present study’s intervention is based on the above premise that there is an association between caregiver and the child’s mental health during admission. Targeting behavioural problems in the caregiver by providing information about admission (to create a feeling of control) and creating avenues for playful interaction between caregiver and child (to reduce anxiety associated with admission) is a possible avenue to prevent mental health problems in children after admission for severe malaria. In this study however, the intervention was not associated with improved mental health outcomes in the children 6 months after discharge. The same intervention has been used in children admitted in intensive care and was associated with fewer mental health problems in children and their caregivers [10, 12]. Some effects were observed at 6 months, while others were observed at 12 months. The 6-month follow-up in this study may have been too short to observe any effect. Alternatively, an intervention developed for children in a US intensive care unit may not translate into an effective intervention for the very different and more resource-limited hospital setting for severe illness in a Uganda hospital, even with adaptation for the Ugandan context.

The evaluation of behaviour was also different, with the present study using the SDQ (primary) and the CBCL (secondary), while the US intensive care unit study used the Behaviour Assessment System for Children (BASC) [12]. The SDQ assesses primarily emotional, conduct, hyperactivity, peer, and prosocial problems, while the BASC version evaluates externalizing problems, internalizing problems, behavioural symptoms, and adaptive skills [12]. Differences in areas being evaluated may have contributed to the differences in study findings, and future studies may need to assess adaptive skills that are tested in BASC but not in SDQ. In addition, in the study of COPE in US ICUs, the BASC scores for the control group were variable over time and increased (more behavioural problems) substantially from 6 to 12 months. This could reflect variability in response to a questionnaire over time, or might reflect increased behavioural problems 12 months after illness. If the latter was the primary driver for differences, then testing at 12 months in this cohort may have revealed problems not found at 6 months follow-up. Finally, malaria is uncommon in the US, and it is unlikely that any child had malaria (11% were admitted for infections like sepsis and meningitis, as reported by Melnyk et al*.* [10]). Diseases can affect behaviour in different ways, so it is possible that the COPE intervention is less effective for children with severe malaria. Countering this is that children in the US COPE ICU study were admitted with many different underlying diagnoses, yet appeared to have a benefit at 12 months after their illness from the COPE intervention. The COPE intervention has been used in other populations, including premature neonates [31] and children with neurological problems [32], with some success in improved mental health for caregiver, child or both, so it will be important to determine if in longer term follow-up better outcomes are seen with the intervention in children with severe malaria.

The study’s inability to completely conceal the interventions participants received on the ward may have resulted in bias as caregivers rated their children’s behavioural problems. Children in the intervention received different play activities from the control group. This bias in reporting may have affected the intervention, resulting in no differences in outcomes between the groups.

The present study was limited by its short follow-up duration of 6 months which could have resulted in no effect seen at that time point. Additionally, there was a higher rate of loss to follow-up in the intervention arm leading to a smaller sample size, which limited the power of the study. It was not possible to separate participants from the different treatment arms on the ward, which may have led to caregivers noticing different interventions being given to their child. The strengths of the study include its randomized design and blinding of the assessors that limits bias in assessing the outcomes.

This study’s behavioural intervention had no effect on children’s mental health problems 6 months after discharge. There is a need to identify other behavioural interventions that could improve mental health outcomes for children admitted for severe malaria in this setting to prevent long-term consequences in adulthood, such as mental health problems and challenges with employment, education and social life [5, 6]. A prior study in Uganda identified neurologic deficits and seizures during admission as being associated with these behavioural problems [3]. In addition to behavioural interventions, adjunctive therapy to improve outcome after severe malaria may potentially lead to improved mental health outcomes, and could supplement behavioural interventions [33].

## Supplementary Information

**Additional file 1.** Narrative script for the control group.

**Additional file 2.** Narrative script for the intervention group.

**Additional file 3.** Generalized estimating equations comparing outcomes between the two groups.

## Data Availability

The datasets used and/or analysed during the current study are available from the corresponding author on reasonable request.

## Abbreviations

BASC: Behaviour Assessment System for Children
CBCL: Child Behaviour Checklist
COPE: Creating opportunities for parent empowerment
GEE: Generalized estimating equations
HSCL: Hopkins Symptom Checklist
ICU: Intensive care unit
PTSD: Post-traumatic stress disorder
SDQ: Strengths and Difficulties Questionnaire

## References

[1] WHO (2018). World malaria report.

[2] Dondorp AM, Fanello CI, Hendriksen ICE, Gomes E, Seni A, Chhaganlal KD, Bojang K (2010). Artesunate versus quinine in the treatment of severe falciparum malaria in African children (AQUAMAT): an open-label, randomised trial. Lancet.

[3] Idro R, Kakooza-Mwesige A, Asea B, Ssebyala K, Bangirana P, Opoka RO (2016). Cerebral malaria is associated with long-term mental health disorders: a cross sectional survey of a long-term cohort. Malar J.

[4] Ssenkusu JM, Hodges JS, Opoka RO, Idro R, Shapiro E, John CC (2016). Long-term behavioral problems in children with severe malaria. Pediatrics.

[5] Copeland WE, Wolke D, Shanahan L, Costello EJ (2015). Adult functional outcomes of common childhood psychiatric problems: a prospective, longitudinal study. JAMA Psychiatry.

[6] Fergusson DM, Horwood LJ, Ridder EM (2005). Show me the child at seven: the consequences of conduct problems in childhood for psychosocial functioning in adulthood. J Child Psychol Psychiatry.

[7] Nakitende AJ, Bangirana P, Nakasujja N, Semrud-Clikeman M, Ssemata AS, John CC, Idro R (2018). “I feel so bad but have nothing to do.” Exploring Ugandan caregivers’ experiences of parenting a child with severe malaria and subsequent repeated uncomplicated malaria. Malar J.

[8] Davydow DS, Richardson LP, Zatzick DF, Katon WJ (2010). Psychiatric morbidity in pediatric critical illness survivors: a comprehensive review of the literature. Arch Pediatr Adolesc Med.

[9] Rennick JE, Rashotte J (2009). Psychological outcomes in children following pediatric intensive care unit hospitalization: a systematic review of the research. J Child Health Care.

[10] Melnyk BM, Crean HF, Feinstein NF, Fairbanks E, Alpert-Gillis LJ (2007). Testing the theoretical framework of the COPE program for mothers of critically ill children: an integrative model of young children’s post-hospital adjustment behaviors. J Pediatr Psychol.

[11] Durkin MS, Davidson LL, Desai P, Hasan ZM, Khan N, Shrout PE (2005). Validity of the ten-question screen for childhood disability: results from population based studies in Bangladesh, Jamaica and Pakistan. Epidemiology.

[12] Melnyk BM, Alpert-Gillis L, Feinstein NF, Crean HF, Johnson J, Fairbanks E (2004). Creating opportunities for parent empowerment: program effects on the mental health/coping outcomes of critically ill young children and their mothers. Pediatrics.

[13] Goodman R (2001). Psychometric properties of the strengths and difficulties questionnaire. J Am Acad Child Adolesc Psychiatry.

[14] Goodman R (1997). The strengths and difficulties questionnaire: a research note. J Child Psychol Psychiatry.

[15] Okello J, Onen T, Misisi S (2007). Psychiatric disorders among war-abducted and non-abducted adolescents in Gulu district, Uganda: a comparative study. Afr J Psychiatry.

[16] Kinyanda E, Kizza R, Abbo C, Ndyanabangi S, Levin J (2013). Prevalence and risk factors of depression in childhood and adolescence as seen in 4 districts of North-Eastern Uganda. BMC Int Health Hum Rights.

[17] Kashala E, Elgen I, Sommerfelt K, Tylleskar T (2005). Teacher ratings of mental health among school children in Kinshasa, Democratic Republic of Congo. Eur Child Adolesc Psychiatry.

[18] Achenbach TM, Rescorla LA (2000). Manual for the preschool forms & profiles: an integrated system of multi-informant assessment.

[19] Achenbach TM, Rescorla LA (2000). Manual for the ASEBA preschool forms & profiles.

[20] Bangirana P, Nakasujja N, Giordani B, Opoka RO, John CC, Boivin MJ (2009). Reliability of the Luganda version of the child behaviour checklist in measuring behavioural problems after cerebral malaria. Child Adolesc Psychiatry Ment Health.

[21] Ivanova M, Achenbach T, Dumenci L, Rescorla L, Almqvist F, Weintraub S (2007). Testing the 8-syndrome structure of the child behavior checklist in 30 societies. J Clin Child Adolesc Psychol.

[22] Derogatis LR, Lipman RS, Rickels K, Uhlenhuth EH, Covi L (1974). The Hopkins symptom checklist (HSCL). A measure of primary symptom dimensions. Mod Probl Pharmacopsychiatry.

[23] Iwata M, Han S, Hays R, Doorenbos AZ (2019). Predictors of depression and anxiety in family members 3 months after child’s admission to a pediatric ICU. Am J Hosp Palliat Care.

[24] Ashaba S, Kakuhikire B, Vořechovská D, Perkins JM, Cooper-Vince CE, Maling S (2018). Reliability, validity, and factor structure of the Hopkins symptom checklist-25: population-based study of persons living with HIV in rural Uganda. AIDS Behav.

[25] Bangirana P, John CC, Idro R, Opoka RO, Byarugaba J, Jurek AM, Boivin MJ (2009). Socioeconomic predictors of cognition in Ugandan children: implications for community interventions. PLoS ONE.

[26] John CC, Bangirana P, Byarugaba J, Opoka RO, Idro R, Jurek AM (2008). Cerebral malaria in children is associated with long-term cognitive impairment. Pediatrics.

[27] Durkin MS, Hasan ZM, Hasan KZ (1995). The ten questions screen for childhood disabilities: its uses and limitations in Pakistan. J Epidemiol Community Health.

[28] Kakooza-Mwesige A, Ssebyala K, Karamagi C, Kiguli S, Smith K, Anderson MC (2013). Adaptation of the ‘ten questions’ to screen for autism and other neuro-developmental disorders in Uganda. Autism.

[29] Shears D, Nadel S, Gledhill J, Garralda ME (2005). Short-term psychiatric adjustment of children and their parents following meningococcal disease. Pediatr Crit Care Med.

[30] Shears D, Nadel S, Gledhill J, Gordon F, Garralda ME (2007). Psychiatric adjustment in the year after meningococcal disease in childhood. J Am Acad Child Adolesc Psychiatry.

[31] Oswalt KL, McClain DB, Melnyk B (2013). Reducing anxiety among children born preterm and their young mothers. MCN Am J Matern Child Nurs.

[32] Duffy LV, Vessey JA (2016). A randomized controlled trial testing the efficacy of the creating opportunities for parent empowerment program for parents of children with epilepsy and other chronic neurological conditions. J Neurosci Nurs.

[33] John CC, Kutamba E, Mugarura K, Opoka RO (2010). Adjunctive therapy for cerebral malaria and other severe forms of *Plasmodium falciparum* malaria. Expert Rev Anti Infect Ther.

