# Supplementary material for: Use of the creating opportunities for parent empowerment programme to decrease mental health problems in Ugandan children surviving severe malaria: a randomized controlled trial
Source: Malar J. 2021 Jun 13;20:267. doi: 10.1186/s12936-021-03795-y (PMC8201864; doi:10.1186/s12936-021-03795-y)
Supplement: Supplementary file 1 — Additional file 1. Narrative script for the control group. [file 12936_2021_3795_MOESM1_ESM.docx]

**Control group**

**Phase I (when in Acute care unit)**

Before providing the information in the control group, it is important to build rapport with the child and the parent/caretaker i.e have introductions and purpose to this information. After gaining rapport then proceed delivering the following information

1. *When you bring the child to hospital the doctor will ask some questions about the child's illness like, what the symptoms are, whether you took medicine elsewhere, how your child has been growing and other questions about the child*
2. *The doctor will then conduct a clinical examination on the child.*
3. *The doctor will order for laboratory tests in order to manage the child better.*
4. *The doctor will prescribe treatment which will be administered on the ward.*
5. *Your child may require supportive care like feeding through a nasal tube or Oxygen therapy as the clinician may have assessed during the clinical examination.*
6. *The Acute Care Unit is the first point of treatment and it is a one day stay before you are transferred to other wards depending on the admitting day and condition of the child.*
7. *To avoid breakage, adults are advised not to sleep on the child’s bed.*
8. *The medical staff will continue to monitor the status of the child while on the ward.*
9. *Ward rounds are conducted every week where a senior doctor and a number of other health providers interact with you and the child to monitor progress for better management. You are required to cooperate and not hide your child.*
10. *We have a number of different healthcare professionals in this place. i.e. Doctors, nurses, laboratory providers and support staff e.g. cleaners. It is important that you respect each one of them in order to be served best.*
11. *You will find different caregivers to children in the wards; take full responsibility of your belongings and child. At the same time respect other caregivers; incase of any grievance seek assistance from the nurse in charge of the ward.*
12. *Each ward had toilet facilities, avoid disposing pampers and other items in these place to avoid blockage of the drainages.*
13. *The hospital provides breakfast and meals for you and your child. This does not stop you from bringing food of your choice.*
14. *The child’s file is kept under the custody of the nurse in charge of the ward. It is not for you to carry away.*
15. *At the time of discharge, a discharge form will be handed to you showing what medicine to take and when to return if necessary..*
16. *In case you have not received all the prescribed medicine from the pediatric ward pharmacy, you will be required to buy it outside of the hospital facility from a pharmacy.*
17. *Make sure you supervise the child’s taking of the medicine.*
18. *It is important that you bring back the child for review on that date together with the discharge medical form. However, in the event that a situation arises before the review date, bring back the child for further medical.*
19. *Thank you for taking care of your child and we wish your child a good recovery.*

**Phase IIa (When on the general ward)**

Summarize the above information again

**Phase IIb**

The parent or caregiver will read a story to the child not related to hospitalization.

Some of the stories will include;

- Baa baa black sheep
- Book of animals
- An activity book with pictures to be shaded (parent-child engagement) plus crayons.

**Phase III (at home after discharge)**

The psychologist calls the caregiver and informs them of the need to contact their doctor is the child develops any emotional or behavioral problems.
