# Supplementary material for: Use of the creating opportunities for parent empowerment programme to decrease mental health problems in Ugandan children surviving severe malaria: a randomized controlled trial
Source: Malar J. 2021 Jun 13;20:267. doi: 10.1186/s12936-021-03795-y (PMC8201864; doi:10.1186/s12936-021-03795-y)
Supplement: Supplementary file 2 — Additional file 2. Narrative script for the intervention group. [file 12936_2021_3795_MOESM2_ESM.docx]

**Intervention Group**

**Phase I, within 6 to 16 hours of admission** (Information about child’s likely emotional reaction during admission in hospital).

Read the italicized section below to the caregiver/parent:

*Most of the time when children are brought to the hospital either for admission or as out- patients no matter the precautions initially taken, they will get scared. Children hear and see different things that cause them to become scared. The hospital may also cause the parent or caregiver to get scared. Some of these reactions are brought on by the way treatment is given like needle pricks; need to have a cannula inserted or tube in the mouth. Other things like crying and screaming infants; health providers’ clothing; stethoscopes and the strange arrangement of beddings in the hospital setting may create fear in the child and caregiver.*

*Some of the reactions that children may exhibit may include the following:*

***Emotional reactions***

- *Fear*
- *Worry*
- *Scared*
- *Teary eyes*
- *Startled*
- *Does not respond to sound of your/health provider’s voice*
- *Increased medical fears*
- *Anger*

***Behavior reactions***

- *Endless crying*
- *Screaming*
- *Withdrawal*
- *Temper tantrums*
- *Sleeplessness*
- *Frequent awakening*
- *Irritability*
- *Avoiding situational reminders e.g. healthcare providers, hospital facility.*
- *Fear of being in a closed room*
- *Showing readiness to run out of examination room*
- *Regression in once attained developmental milestones.*
- *Aggression towards caregiver, parent of health provider.*
- *Lack of cooperation*
- *Failure to become quiet when soothed after medical procedures.*
- *Pushing or slapping at healthcare provider or caregiver.*
- *Attempts to run and fight with caregiver or healthcare provider.*
- *Become demanding in their needs and wants.*
- *Begin to speak like a baby*
- *Does not engage in play*

**Phase II, within 2 to 16 hours after transfer to the general ward** (Caregivers/mothers are provided with suggestions to enhance coping outcomes in their children)

**Recap the above emotional and behavioral problems then read the italicized section below:**

*These are normal reactions that children express. You may too feel the same when you visit a hospital. These emotional and behavior reactions are the way through which a child gets to communicate to us adults*.

**As you speak with the caregiver, allow them to express how they would cope with their child’s emotional and behavior reactions in a hospital facility and at post discharge.**

Include the following;

- Don’t slap or abuse the child otherwise this worsens the situation.
- Sing with or to the child to calm them down and distract their attention away from the medical procedure to be conducted.
- Be present
- Speak with/to the child.
- What comforts the child when she/he is upset e.g a doll
- Ask the child to speak clearly so you can hear what they are saying. This will help to stop the behavior before you leave hospital.
- Allow the child to play in hospital which creates a sense of normality to the initially feared environment.
- Initiate the play to help you understand the child understands of the hospital situation.

**Phase IIb** (Three activities for parent-child interaction completed before discharge)

Normative play

1. For 1.5 to 2 years old; pretend play using dolls to encourage the child to express emotions in a hospital environment
2. For 3 to 4 years; stacking blocks

Medical – therapeutic play

1. For 1.5 to 2 years old; Use of medical items in play like hospital dolls (of nurses/doctors, father, mother, girl, boy, thermometer, ambulance, baby bottle, band aids, scissors, medical art, medicine bottles.
2. For 3 to 4 years; Colouring pictures of doctors and nurses
3. For 3 to 4 years; Story of a child in hospital

**Phase III**

The caregiver will be contacted on phone 2-3 days post discharge. The following 5 minute script will be read;

*After discharge, some children may show behavior resulting from their hospitalization. Some of these behaviors include;*

- 1. *reduced self esteem*
  2. *decreased self confidence*
  3. *Refusal to take the medicine*
  4. *Withdrawn*
  5. *Speaks negatively about the hospital experience*
  6. *Role plays the hospital experience*

*These are normal behaviors given the experience they had during hospitalization. As a parent or caregiver, it is not good to react in anger towards the child or punish them for this behavior. Some things you can do to help the child adjust include;*

1. *Cuddling the child*
2. *Showing more care and support for the child*
3. *Reading stories with a happy theme*
4. *Playing with the child*
