# Supplementary material for: Use of the creating opportunities for parent empowerment programme to decrease mental health problems in Ugandan children surviving severe malaria: a randomized controlled trial
Source: Malar J. 2021 Jun 13;20:267. doi: 10.1186/s12936-021-03795-y (PMC8201864; doi:10.1186/s12936-021-03795-y)
Supplement: Supplementary file 3 — Additional file 3. Generalized estimating equations comparing outcomes between the two groups. [file 12936_2021_3795_MOESM3_ESM.docx]

**Additional file 4 to:** Bangirana, P, et al. Use of the Creating Opportunities for Parent Empowerment program to decrease mental health problems in Ugandan children surviving severe malaria: a randomized controlled trial

**Methods and Results**

Emotional and behavioral outcome measurements were conducted at admission before the intervention and 6 months after discharge. We compared outcome measurements between the intervention and control groups using the Generalized Estimating Equations (GEE) with robust standard errors and assuming an exchangeable correlation structure between the measurements at the two time points. There were no differences in scores between the intervention and control groups.

**Table S1. Mean differences of child and caregiver emotional and behavioral outcomes using all time-point measurements**

| **Domain** | **Intervention (n=45)** | **Control (n=50)** | **Mean difference (95% CI)** | **P value** |
| --- | --- | --- | --- | --- |
| SDQ Total problems | 13.14 (0.48) | 13.34 (0.48) | -0.20 (-1.53 to 1.14) | 0.77^1^ |
| SDQ Emotional problems | 2.33 (0.21) | 2.38 (0.22) | -0.05 (-0.64 to 0.54) | 0.86^1^ |
| SDQ Conduct problems | 3.13 (0.20) | 3.46 (0.19) | -0.33 (-0.87 to 0.21) | 0.23^1^ |
| SDQ Hyperactivity problems | 3.78 (0.17) | 3.91 (0.18) | -0.13 (-0.62 to 0.36) | 0.60^1^ |
| SDQ Peer problems | 3.81 (0.15) | 3.53 (0.15) | 0.29 (-0.13 to 0.70) | 0.18^1^ |
| SDQ Prosocial problems | 7.98 (0.20) | 7.98 (0.18) | -0.004 (-0.54 to 0.53) | 0.99^1^ |
| CBCL Total Problems | -1.06 (0.07) | -1.10 (0.07) | 0.04 (-0.15 to 0.23) | 0.69^1^ |
| CBCL Internalizing Problems | -1.13 (0.06) | -1.16 (0.06) | 0.04 (-0.13 to 0.20) | 0.68^1^ |
| CBCL Externalizing Problems | -1.26 (0.06) | -1.22 (0.07) | -0.04 (-0.22 to 0.14) | 0.66^1^ |
| HSCL Caregiver anxiety | 4.66 (0.53) | 5.21 (0.61) | -0.54 (-2.12 to 1.03) | 0.50^2^ |
| HSCL Caregiver depression | 11.29 (0.88) | 10.89 (0.98) | 0.40 (-2.16 to 2.97) | 0.76^2^ |

All estimates are adjusted means (standard error) unless otherwise stated. Intervention and control groups mean scores were compared between the intervention and control groups using Generalized Estimating Equations (GEE) with robust standard errors.

^1^Adjusted for caregiver depression, mother’s education and child’s sex

^2^Adjusted for presence of diarrhea and behavioral problems for the child during admission.
